# Perinatal outcomes of spontaneous single fetal death in monochorionic twin pregnancies: a single-center retrospective study

**DOI:** 10.3389/fmed.2025.1646852

**Published:** 2025-10-27

**Authors:** Chunyan Deng, Shiyu Chen, Qing Hu, Guiqiong Huang, Xiaodong Wang, Haiyan Yu

**Affiliations:** ^1^Department of Obstetrics and Gynecology, West China Second University Hospital, Sichuan University, Chengdu, China; ^2^Key Laboratory of Birth Defects and Related Diseases of Women and Children (Sichuan University), Ministry of Education, Chengdu, China; ^3^Department of Ultrasound, West China Second University Hospital, Sichuan University, Chengdu, China

**Keywords:** monochorionic twin pregnancy, spontaneous single intrauterine fetal demise, prenatal outcomes, neonatal cerebral damage, twins

## Abstract

**Objective:**

The aim of this study was to investigate the perinatal outcomes of surviving co-twins after spontaneous single intrauterine fetal death (sIUFD) in monochorionic (MC) twin pregnancies and to assess the risk of cerebral injury.

**Methods:**

This retrospective study was conducted on 90 MC twin pregnancies resulting in spontaneous single intrauterine fetal death at our tertiary hospital between January 2012 and December 2023. When termination of pregnancy (TOP) and co-twin deaths were excluded, the patients were subdivided into two groups according to their gestational age (GA) at single fetal death: Group 1, < 24 weeks; Group 2, ≥24 weeks.

**Results:**

There were 83 MCDAs and seven MCMAs. Among the patients, the gestational age (GA) at single fetal death was 24.0 ± 5.95 weeks (range 14–34+4 weeks), whereas two patients with co-twin late death, 19 patients with TOP, and 69 patients continuing pregnancy (including seven monochorionic monoamniotic twins) were included. Kaplan–Meier survival curve indicated that the risk of neurological damage and/or fetal anemia increases with GA at sIUFD. Meanwhile, ROC curve showed that GA at sIUFD is a predictor of cerebral lesions, and the lowest risk of cerebral damage for the co-twin occurred before 25+6 weeks of gestation. Among the 69 continuing pregnancies, the delivery age was 35.1 ± 3.3 weeks, with 68 live births, and the birth weight was 2,355.0 ± 681.0 g. The rates of preterm birth and NICU admission were 62.3 and 50.7%, respectively. Cerebral injury was detected in 10 neonates, three newborn deaths due to cerebral damage caused by extreme prematurity, and two infants (2.9%) with neurological abnormalities. The neonatal outcomes were compared between GAs at single fetal death < 24 weeks (Group 1, 34 cases) and those at ≥24 weeks (Group 2, 35 cases). The average gestational age at delivery (36.6 ± 3.3 vs. 33.7 ± 2.5, *p* = 0.000) and birth weight (2,639.5 ± 705.2 g vs. 2,074.9 ± 546.9 g, *p* = 0.000) were greater in the Group 1. The incidence rates of extreme preterm, late preterm, live birth and neonatal mortality were not significantly different between the two groups. The gestational age of patients with sIUFD was negatively correlated with delivery age, with a Spearman's rho = −0.588^**^ (*p* = 0.000).

**Conclusions:**

GA at sIUFD is a predictor of cerebral lesions, the greater the gestational age of sIUFD is, the higher the risk of brain injury among surviving co-twins. There was a negative correlation between gestational age at sIUFD and delivery age in this study. The prenatal outcomes of surviving co-twin with sIUFD before 24 weeks were better than those after 24 weeks

## Introduction

The incidence of twin pregnancies has increased in recent decades due to advanced maternal age and the widespread application of artificial reproductive procedures. Compared with dichorionic (DC) or singleton pregnancies, monochorionic (MC) twin pregnancies are associated with increased perinatal mortality and morbidity (1). Neurological morbidities are more common in monochorionic twins than in dichorionic gestations (2–4). This phenomenon is related mainly to placental architecture and vascular anastomoses in the MC placenta. This may lead to hypoperfusion of the surviving twin at the time of death. Up to 20% of surviving twins might suffer adverse neurodevelopmental outcomes (3, 5). The rate of single intrauterine fetal death (sIUFD) is higher in pregnancies with monochorionic twins (7.5%), than in those with dichorionic twins (3%) (6). However, a single death may occur in twin pregnancies at any gestation. Deaths that occur in the first trimester usually lead to fewer complications than deaths that occur in the second and third trimesters (2). Single fetal death during twin pregnancy in the second- and third-trimesters is reported to increase the incidence of death, preterm birth, and neurological injury in the surviving co-twins (7, 8).

There are controversies related to the close follow-up, including the frequency of ultrasound scans, neurosonography and/or magnetic resonance imaging (MRI). Prenatal identification of brain abnormalities in the surviving co-twin is essential for parental counseling. Preterm birth is also a main condition that causes cerebral damage in newborns; moreover, it is well-known that sIUFD in MC twins is associated with a high risk of both iatrogenic and spontaneous preterm delivery.

The aim of the current study was to investigate the clinical outcomes of monochorionic twin pregnancies in cases of spontaneous sIUFD and to perform subgroup analyses of the prenatal outcomes based on the gestational age (GA) at which a single fetus dies in surviving co-twins.

## Materials and methods

### Study population

A retrospective descriptive study was designed, involving all MC pregnancies with sIUFD after 14 weeks were diagnosed or referred to the Fetal Medicine Unit of the West China Second University Hospital, a large tertiary referral center, between January 2012 and December 2023. This study was approved by the Ethics Committee of West China Second University Hospital (no.2023-272). Monochorionicity was confirmed by ultrasound at 11–13+6 weeks of gestation (presence of the T sign) (9, 10). Twin pregnancies were excluded from the study if the antenatal ultrasound showed twin reversed arterial perfusion (TRAP) sequence and or treated by intrauterine therapeutic procedures (fetoscopic selective laser ablation of placental vascular anastomoses or umbilical cord occlusion). These pregnancies were followed-up and delivered at our center.

Data on maternal and pregnancy characteristics (maternal age, conception method, GA at sIUFD), the presence and type of complications and gestational age at single fetal death were collected. Data on pregnancy complications (cerebral damage in the surviving co-twin, the couple who refused to continue pregnancy, co-twin late death, preterm delivery) and neonatal outcomes (GA at birth, type of delivery, birthweight, and Apgar score) were also recorded.

### Protocol study and follow-up

TTTS was defined by an oligo-polyhydramnios sequence according to the guidelines for the maximum vertical pocket of amniotic fluid in the recipient and donor (≥8 cm before 20 weeks of GA and ≥10 cm after 20 weeks GA in the recipient and < 2 cm in the donor) (11); s-IUGR was defined by the presence of one twin with an estimated fetal weight < 10th percentile and an intertwin estimated fetal weight >25% and was classified according to the pattern of end-diastolic velocity blood flow in the umbilical artery (11, 12).

Assessment of survival co-twin included Doppler measurement of the middle cerebral artery-peak systolic velocity (MCA-PSV) and detailed neurosonography as soon as single fetal death was detected to identify fetal anemia and to evaluate signs of ischemic/hemorrhagic cerebral lesions (13, 14). MCA-PSV greater than 1.5 multiples of the median (MoM) was used as a screening test to identify anemic fetuses (15). Fetal MRI was offered in all cases at least 3 weeks after sIUFD or if there was suspected brain damage on ultrasound (16, 17).

Cerebral damage included abnormal neuroimaging findings on ultrasound and/or MRI both prenatally and/or postnatally. Severe cerebral damage was defined as at least one of the following: intraventricular hemorrhage ≥grade III, cystic periventricular leukomalacia ≥grade II, arterial or venous infarction, and porencephalic cyst, basal ganglia, thalamic and/or cortical hypoxic–ischemic lesions (18). When a prenatal cerebral injury was detected, parents were extensively counseled by a multidisciplinary team regarding possible perinatal outcomes before making any decision [to continue pregnancy or to choose for termination of pregnancy (TOP)]. Late termination of pregnancy may be considered when serious neurological harm is diagnosed (19). Late termination is an option in some countries (include China) where local policies allow this procedure.

Cranial ultrasonography is convenient but lacks sensitivity for the evaluation of the nature and extent of brain injury. Brain MRI should be performed for neonates suspected of hypoxic—ischemic encephalopathy, intracranial infection, stroke, and unexplained convulsions. As for extremely preterm or extremely low-birth-weight infants without abnormal ultrasound findings, it is recommended that they undergo an MRI examination at term—equivalent age once (20). Two MRI or magnetic resonance spectroscopy scans will assist with full delineation of the nature and extent of cerebral injury. The first between 24 and 96 h of life with emphasis on the evaluation of diffusion and spectroscopic abnormalities to assist in clinical management and evaluation of the timing of cerebral injury, and a second at day 10 of life or later (21). In this study, for those newborns who were transferred to the neonatal intensive care units (NICU), at least two cranial ultrasound or MRI scans were performed. Severe cerebral damage on postnatal ultrasound or MRI was defined by the use of the same imaging criteria as those uesd in prenatal neuroimaging. Postnatal eurodevelopment was closely assessed by pediatric neurologists.

The ISUOG Practice Guidelines (12) recommend first-trimester screening for chromosomal anomalies and an anatomic scan at 18–22 weeks. In accordance with Chinese national guidelines, ultrasound scans of fetal structures were completed at 18–24 weeks (22). In a study by McPherson et al. reported the risk of co-twin demise is the highest when the first twin demise is less than 24 weeks and lowest when greater than 28 weeks (23). Therefore, pregnancies that continued pregnancy were divided into two groups according to GA at sIUFD: Group 1 (< 24 weeks) and Group 2 (≥24 weeks). On the basis of delivery GA, preterm birth was subdivided into four stages: extremely preterm (24–27+6 weeks), very preterm (28–31+6 weeks), moderately preterm (32–33+6 weeks) and late preterm (34–36+6 weeks) (24). High rates of neonatal morbidity and mortality have been observed in very preterm infants born before 32 weeks of gestation (25).

### Statistical analysis

The data were analyzed using the Statistical Package for Social Sciences (SPSS) version 25.0 (2017, SPSS Inc., Chicago, IL, US). Data are presented as the mean ± standard deviation (SD), median (interquartile range) or number (%). Continuous variables were compared using the Student's *t* test (for normally distributed data) or the Mann–Whitney *U* test (for non-normally distributed data). Categorical type outcomes were analyzed using the chi-square test or Fisher's exact test. A *p* value < 0.05 was considered statistically significant. Spearman's correlation analysis was used to study the relationship between the gestational age at single fetal death and the delivery gestational age of surviving co-twins.

## Results

**Figure 1:**
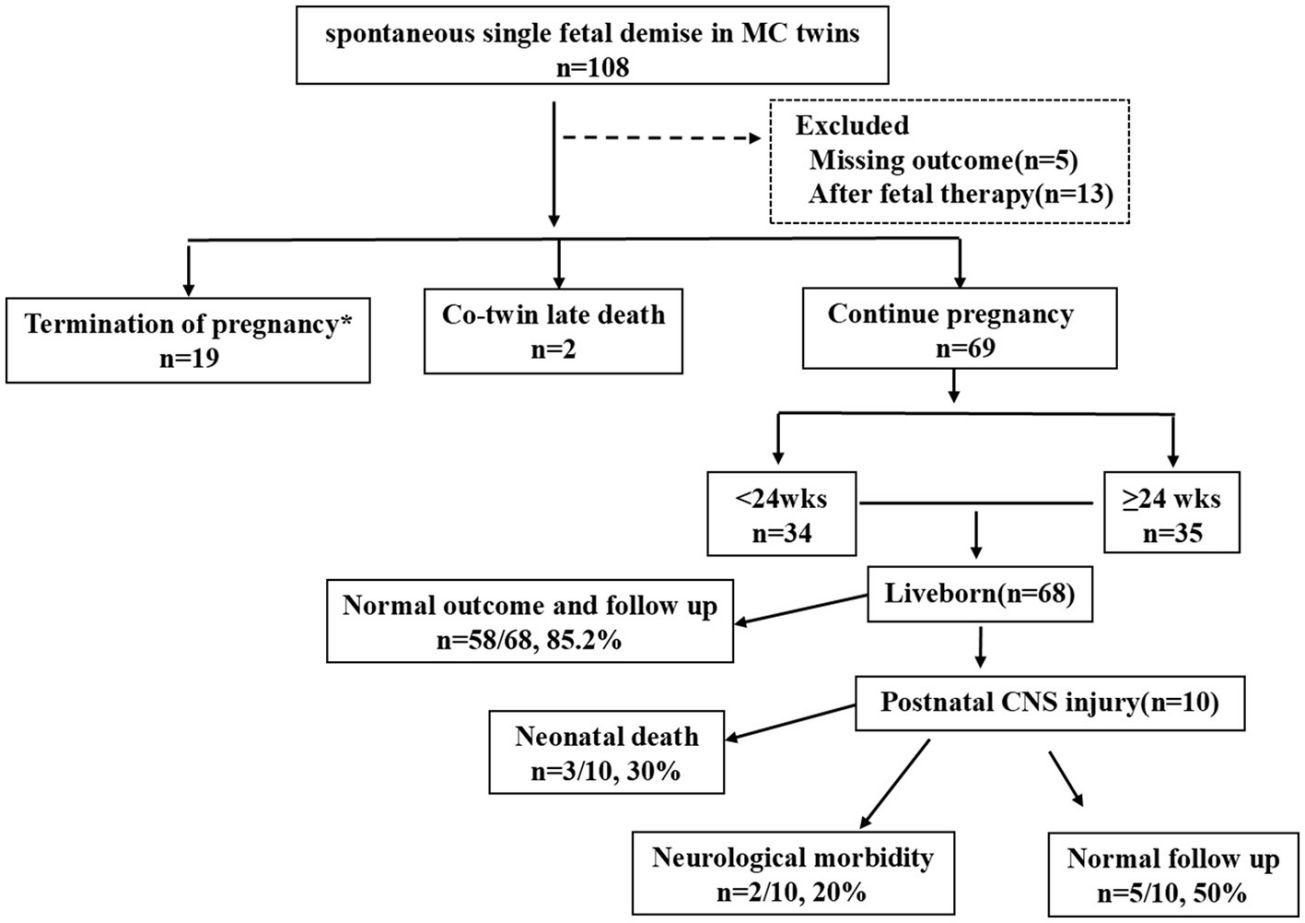
Flowchart of the study population. MC, monochorionic; DC, dichorionic; wks, weeks; CNS, central nervous system.

During the study period, a total of 90 MC pregnancies (83 MCDA pregnancies and seven MCMA pregnancies) with spontaneous sIUFD met the inclusion criteria. Two cases involved late co-twin death, 19 cases involved TOP, and 69 cases involved continuing pregnancies. The included patients are shown in Figure 1.

The details of the antenatal characteristics of the study cohort are shown in Table 1. The GA at single-fetal death was 24.0 ± 5.95 weeks (range 14–34+4 weeks). The complications before sIUFD were as follows: 15 cases (15/90, 16.7%) of discordant malformations, eight cases of sIUGR (8/90, 8.9%), five cases of TTTS (5/90, 5.6%), and five cases of cord entanglement (5/90, 5.6%) in monochorionic monoamniotic (MCMA) twins. The other cases did not present any special complications.

**Table 1:** Characteristics of 90 monochorionic twin pregnancies complicated with spontaneous single fetal death.

**Characteristics**	**MC pregnancies (*n* = 90)**
Maternal age (years)	29.07 ± 4.60
**Conception method**	
ART	15 (16.7%)
Spontaneous	75 (83.3%)
Type of pregnancy
MCDA	83 (92.2%)
MCMA	7 (7.8%)
**Complicated pregnancies before sIUFD**	
TTTS	5 (5.6%)
Discordant malformation	15 (16.7%)
sIUGR	8 (8.9%)
Cord entanglement	5 (5.6%)
Uncomplicated pregnancies	57 (63.3%)
Interval between fetal demise and delivery (days)	63.6 ± 58.9
GA at single fetal death (wks)	24.0 ± 5.95
< 20	26 (28.9%)
20–24	17 (18.9%)
24–28	19 (21.1%)
≥28	28 (31.1%)
Co-twin death	2 (2.2%)
TOP	19 (21.1%)
< 24 wks	7 (7/19, 36.8%)
≥24 wks	12 (12/19, 63.2%)

Values are expressed as n (%), mean (±SD)

ART, assisted reproductive technology; GA, gestational age; TTTS, twin-twin transfusion syndrome; MCDA, monochorionic diamniotic; MCMA, monochorionic monoamniotic; sIUGR, selective intrauterine growth restriction; sIUFD, single uterine fetal death. wks, weeks. TOP, termination of pregnancy.

The GA at sIUFD diagnosis was 24.0 ± 5.95 weeks; in 26 (28.9%) patients; fetal death was detected before 20 weeks, and 28 (31.1%) deaths occurred after 28 weeks of gestation. TOP occurred in 19 cases (21.1%). Among cases with TOP, seven cases were complicated with cerebral damage, as determined by fetal ultrasound and/or MRI. MCA-PSV>1.5 MoM was accompanied by fetal hydrops or abnormal amniotic fluid volume in eight cases, in which the couples refused intrauterine fetal blood transfusion and decided to terminate. One case involved a co-twin suffering from oligoamnios at 20+4 weeks; one case involved giving up a growth-restricted co-twin due to severe maternal preeclampsia at 25+6 weeks; one couple who suffered TTTS stage V opted for TOP because of worries about the risk of continuing the pregnancy; and one co-twin experienced premature rupture of membranes at 25+5 weeks after sIUFD.

**Figure 2:**
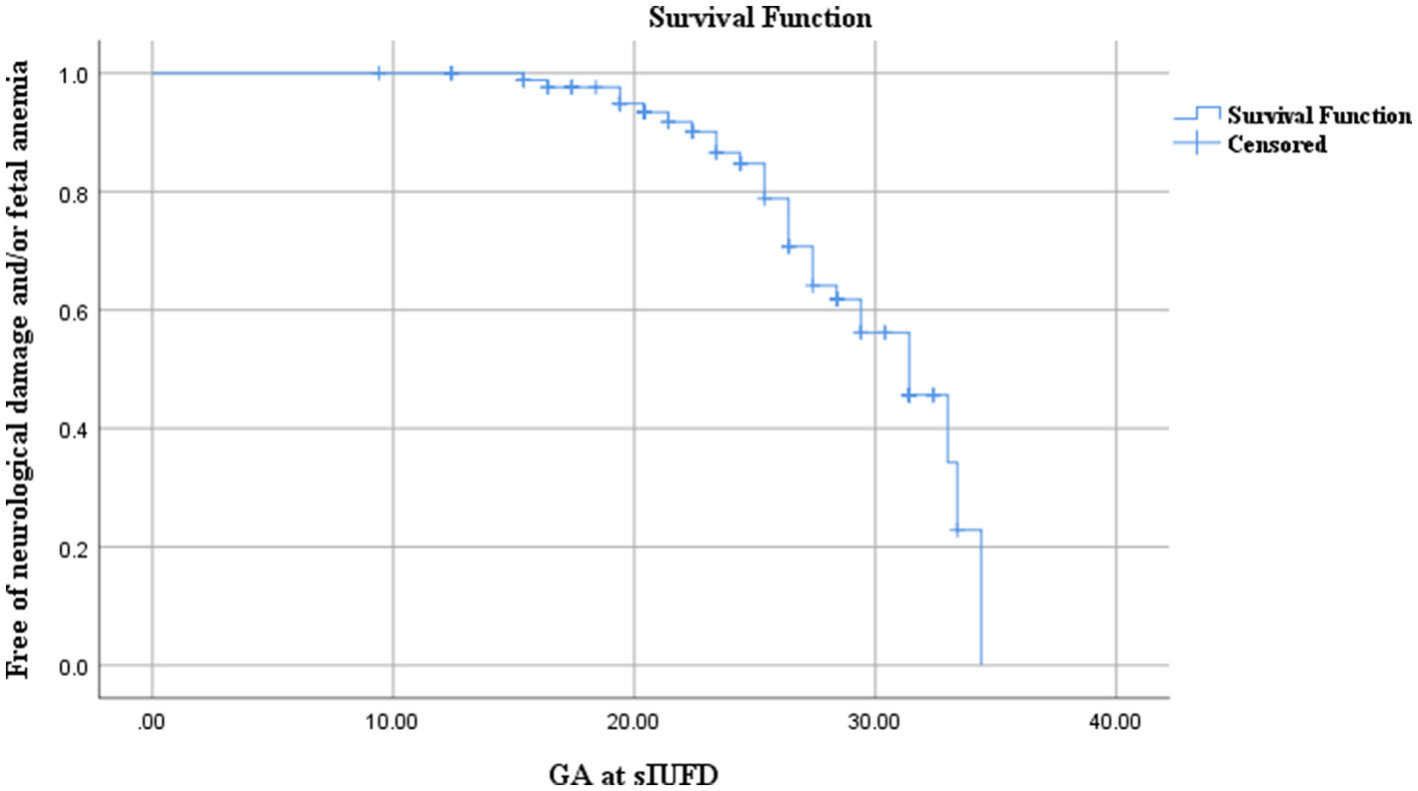
Kaplan–Meier survival curve analysis for the correlation between neurological damage (and/or fetal anemia) and gestational age at sIUFD.

Kaplan–Meier survival curve indicated that the risk of neurological damage and/or fetal anemia increases with GA at sIUFD. With no cases of brain injury (pre/postnatal) or fetal severe anemia when sIUFD was diagnosed before 15+4 weeks of pregnancy, 15.4% (4/26) of cases occurred before 20 weeks, 29.4% (5/17) between 20 and 24 weeks, 31.5% (6/19) between 24 and 28 weeks and 35.7% (10/28) after 28 weeks of gestation (Figure 2).

**Figure 3:**
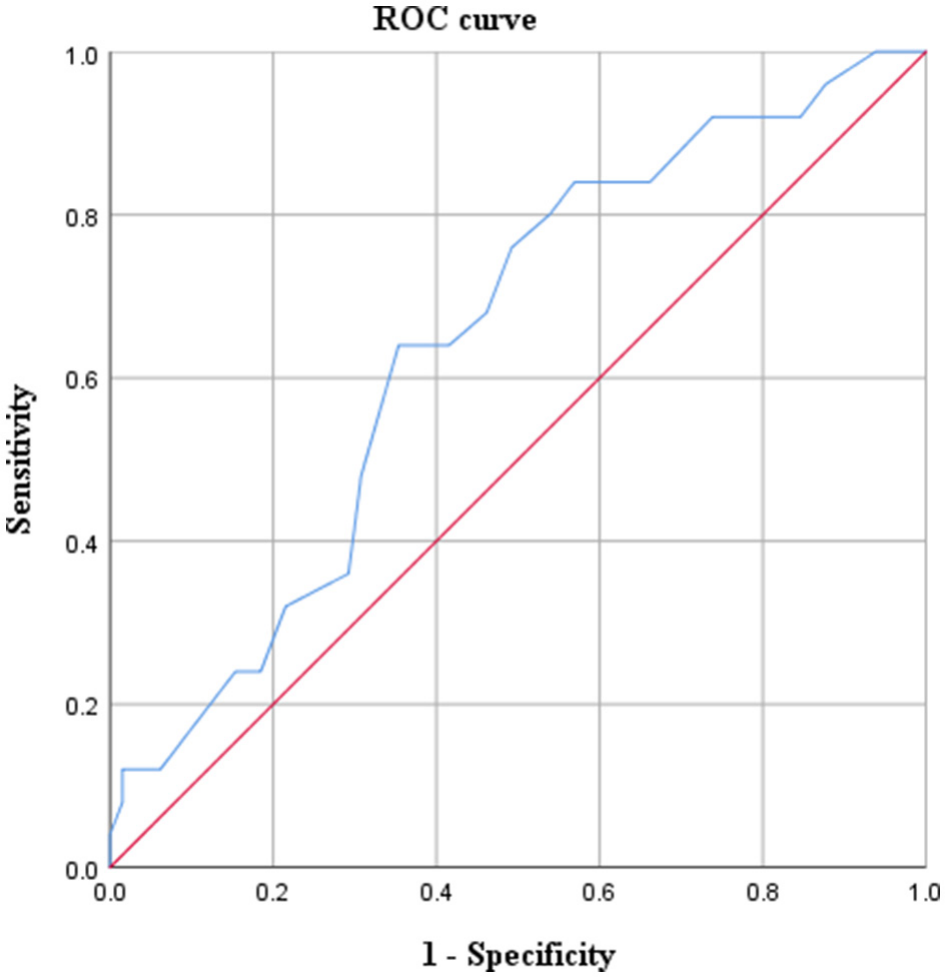
Prenatal prediction of cerebral damage according to gestational age at sIUFD.

ROC curve was used to research the relationship between GA at the sIUFD and cerebral injury (Figure 3). The area under the curve was 0.6446 (*p* = 0.033), and the 95% confidence interval was 0.524–0.768. The Youden index was 0.286, and the lowest risk of cerebral damage for the co-twin occurred before 25+6 weeks of gestation. Thus, GA at sIUFD is a predictor of cerebral lesions.

### Subgroup analysis according to the gestational age of single fetal death in the surviving co-twin

Sixty-nine of the 90 MC pregnancies with sIUFD continued pregnancy, TOP and co-twin deaths were excluded. As shown in Table 2. The average single fetal death time was 24.1 ± 6.2 weeks, and the average delivery time was 35.1 ± 3.3 weeks. The rates of preterm birth and NICU admission were as high as 62.3 and 50.7%, respectively. The average birth weight was 2,355.0 ± 681.0 g. Cerebral injury was detected in 10 neonates, three of whom died of cerebral damage due to extreme prematurity.

**Table 2:** Prenatal outcomes in 69 pregnancies that continued after sIUFD.

**Prenatal outcomes**	**Total (*n* = 69)**	**G1 (*n* = 34)**	**G2 (*n* = 35)**	***p-*value**
GA at fetal death (wks)	24.1 ± 6.2	18.6 ± 3.3	29.4 ± 2.9	0.000
GA at delivery (wks)	35.1 ± 3.3	36.6 ± 3.3	33.7 ± 2.5	0.000
**Preterm delivery**				
24–28 wks, *n* (%)	2 (2.9%)	2 (5.9%)	0	0.145
28–32 wks, *n* (%)	10 (14.5%)	1 (2.9%)	9 (25.7%)	0.007
32–34 wks, *n* (%)	13 (18.8%)	1 (2.9%)	12 (34.3%)	0.001
34–37 wks, *n* (%)	18 (26.1%)	9 (26.5%)	9 (25.7%)	0.943
Term delivery (*n*, %)	26 (37.7%)	21 (61.8%)	5 (14.3%)	0.000
Birth weight (g)	2,355.0 ± 681.0	2,639.5 ± 705.2	2,074.9 ± 546.9	0.000
Live birth, *n* (%)	68 (98.6%)	33 (97.1%)	35 (100%)	0.307
NICU admission, *n* (%)	35 (50.7%)	8 (23.5%)	27 (77.1%)	0.000
Neonatal mortality, *n* (%)	3 (4.3%)	1 (2.9%)	2 (5.7%)	0.572
Postnatal cerebral damage, *n* (%)	10 (14.5%)	2 (5.9%)	8 (22.9%)	0.045

The values are expressed as the means ±SD or n (%).

sIUFD, spontaneous single intrauterine fetal death; GA, gestational age; wks, weeks; g, gram; NICU, neonatal intensive care unit.

There was one case of stillbirth at 24+4 weeks; therefore, 68 live births occurred. Neonatal death within the first 28 days of life occurred in three newborns due to extreme prematurity, with an overall perinatal survival rate of 95.5% (65/68). Abnormal neurological outcomes were detected in two infants (2/68, 2.9%), all of whom had cerebral lesions on neuroimaging.

To compare the outcomes of co-twins at different gestational ages of sIUFD, they were divided into two groups according to GA at sIUFD: Group 1 (< 24 weeks) and Group 2 (≥24 weeks), as shown in Table 2. The mean gestational age at sIUFD was 18.6 ± 3.3 weeks in Group 1, which was significantly earlier than that in Group 2 (29.4 ± 2.9 weeks; *p* = 0.000). In contrast, the mean gestational age at delivery in Group 1 was significantly greater than that in Group 2 (36.6 ± 3.3 vs. 33.7 ± 2.5, *p* = 0.000). Moreover, the birth weights (2,639.5 ± 705.2 g vs. 2,074.9 ± 546.9 g, *p* = 0.000) were greater in Group 1. The incidence rates of extreme preterm birth (24–27+6 weeks), late preterm birth (34–36+6 weeks), live birth and neonatal mortality were not significantly different between Group 1 and Group 2. The rate of term birth (61.8 vs. 14.3%) was higher in Group 1. However, the rates of very preterm (28–31+6 weeks), moderately preterm (32–33+6 weeks), NICU admission and postnatal cerebral damage in Group 2 were higher than those in Group 1.

### Correlation analysis between the age of fetal demise and the delivery age of the participants

**Figure 4:**
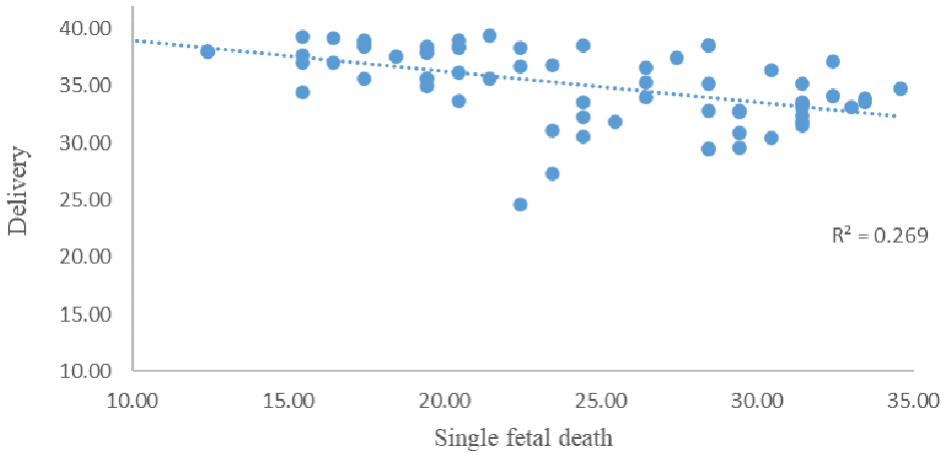
Correlation analysis between age at single fetal death and delivery age.

The correlation between GA at sIUFD and delivery week in 69 continued pregnancy was analyzed via Spearman's correlation analysis (Figure 4). The average single fetal death time was 24.1 ± 6.2 weeks, and the average delivery time was 35.1 ± 3.2 weeks. The correlation coefficient was −0.588^**^ (*p* = 0.000). There was a significant negative correlation between the single fetal death week and the delivery week.

### Prenatal outcomes of MCMA

In this study, seven MCMA pregnancies (7.8%, 7/90) resulted in single fetal death, all of which occurred after 24 weeks of pregnancy (24–31+5 weeks). The birthweight was 1,340–2,440 g. Six neonates had normal outcomes and were healthy. One neonate needed neurorehabilitation therapy. The obstetric outcomes of the seven patients are displayed in Table 3.

**Table 3:** Detailed data of the MCMA twins.

**No**.	**GA of fetal death**	**Interval (days)**	**GA at delivery (wks)**	**Prenatal MRI**	**Type of delivery**	**Birth weight (g)**	**Apgar score (1–5–10 min)**	**Duration of NICU (days)**	**Outcome of surviving co-twin**
1	24	43	30+4	Normal	CS	1,400	8–9–10	55	In good health
2	26+5	53	34	Normal	CS	2,350	9–10–10	10	In good health
3	26+4	62	35+2	Normal	VD	2,440	10–10–10	6	In good health
4	30+3	0	30+3	No	CS	1,390	7–8–9	46	In good health
5	29+4	9	30+6	Normal	CS	1,540	8–9–9	18	In good health
6	31+5	7	32+3	No	CS	1,810	9–9–9	8	In good health
7	29+6	23	32+5	Normal	CS	1,870	5–7–9	23	Need of neurorehabilitation therapy

MCMA, monochorionic monoamniotic twins; CS, cesarean section; VD, vaginal delivery; NICU, Neonatal Intensive Care Unit; GA, gestational age; wks, weeks.

## Discussion

In MC pregnancy, due to the presence of a shared placenta, with dircect intertwin vascular communications, when fetal death occurs in monoamniotic twins, exposes the co-twin to exsanguination and death and risk of neurodevelopmental impairment if it survives (26). Two main theories (“twin embolization syndrome” and “hemodynamic imbalance”) explain the increased mortality and morbidity risk for the surviving co-twin in MC pregnancies. The hemodynamic imbalance theory is widely accepted. However, the risk to the surviving monochorionic cotwin may depend on the type and size of the placental anastomoses. Moreover, the risk of severe cerebral neuroimaging injury in surviving MC co-twin is approximately 20% (3, 5). The risk of death among surviving co-twins due to severe neurological injury ranges from 30 to 50% (3, 27), and an MCA-PSV greater than 1.5 MoM may increase the relative risk of cerebral injury by fivefold (28).

The potential risk of brain damage in the co-twin after sIUFD in MC pregnancies presents diagnostic and management challenges for obstetricians. Moreover, cerebral injury by neuroimaging (MRI and/or ultrasound) does not inevitably imply a long-term developmental delay.

In our cohort, two of 90 (2.2%) pregnancies complicated by spontaneous fetal death resulted in two deaths. Nineteen of 90 (21.1%) patients opted for TOP due to cerebral damage (7/19, 36.8%) or MCA-PSV**>**1.5 MoM accompanied by fetal hydrops or abnormal amniotic fluid volume (8/19, 42.1%). Cerebral injury imaging revealed that in 10 of the 68 liveborn neonates, three newborns died of cerebral damage due to extreme prematurity. Abnormal neurological outcomes were detected in two infants (2/68, 2.9%). In total, in our cohort, the rates of prenatal and postnatal cerebral damage (17/90, 18.9%) were consistent with those reported in the literature (18%−37.5%) (2, 17, 27–29). Additionally, in accordance with Duyos's conclusion (17), our data revealed that GA at sIUFD is a predictor of cerebral lesions in surviving cotwins; the later the GA at s-IUFD is, the higher the risk of brain lesions (*p* = 0.033).

However, immediate delivery when sIUFD occurs far from term worsens the prognosis of the co-twin and must be avoided, as immediate delivery does not protect against cerebral lesions but may contribute to a worse neurological outcome because of prematurity. Antenatal surveillance should be considered in cases for expectant management. Delivery before this would be warranted if there is abnormal fetal testing or other evidence of degenerated placental function. Based on Sondgeroth's report (30): delivery should be considered between 34 and 37 weeks when sIUFD occurs in less than 28 weeks; delivery should be considered between 32 and 34 weeks when sIUFD occurs in 28–34 weeks; delivery should be considered if the demise occurs at or after 34 weeks gestation. More reports have shown that conservative management with close surveillance after sIUFD could be successful as an option to continue the pregnancy until term (17, 31). Healy et al. suggested prolonging the gestational age to 36+6 weeks (27). In our study, the average delivery time was 35.1 ± 3.3 weeks in the cases chosen for continue pregnancy, with satisfactory maternal and fetal outcomes.

In our series, the gestational age at diagnosis of single fetal death was negatively correlated with the gestational age at delivery [Spearman's rho =-0.588^**^ (*p* = 0.000)]. Moreover, the outcomes of the co-twins after sIUFD at different gestational weeks were stratified. The average delivery time was 35.12 ± 3.25 weeks among the 69 women who continued their pregnancies. Furthermore, the mean gestational age at delivery in Group 1 was significantly greater than that in Group 2 (36.6 ± 3.3 vs. 33.7 ± 2.5, *p* = 0.000). The GA at delivery was later than the 33.9 ± 3.9 weeks GA reported by Duyos et al. (17). Moreover, the heavier birthweight (2,355.0 ± 681.0 g) was comparable to that reported by Duyos et al. (17) (2,191 ± 831 g). The average birth weight was greater in Group 1 (2,639.5 ± 705.2) than in Group 2 (2,074.9 ± 546.9; *p* = 0.000).

The rate of preterm birth was 62.3% (43/69), whereas the rate of preterm birth before 34 weeks was 36.2% (25/69). The infants in Group 2 were more likely to have preterm birth between 28 and 34 weeks. High rates of neonatal morbidity and mortality are observed in preterm infants born before 32 weeks of gestation (25). These stratified prenatal outcomes support the view that GA at the IUFD is a predictor of cerebral damage.

MCMA twins are rare, accounting for 5% of all monochorionic pregnancies but < 1% of twins (32). MCMA twins are reported to have high rates of stillbirth and perinatal mortality for a single amniotic cavity with one shared placenta and two umbilical cord entanglements; however, survival rates are improving (33). The rates of livebirth exceed 90% (27). All MCMA twins have a degree of cord entanglement (34, 35). A large survey published in 2019 reported that approximately 3/8 intrauterine fetal deaths were attributed to antenatal hypoxia, which is assumed to be caused by cord entanglement. Duyos et al. reported only two MCMAs from 68 MC pregnancies with sIUFD (17). In our study, seven MCMA patients (7.8%, 7/90) gained survivor co-twins who underwent expectant management following sIUFD, consistent with Liu et al. report (7.6%, 5/66) (36). To release entangled cords and thus decrease the risk of co-twin loss, Greimel reported on umbilical cord transection using fetoscopic grasping forceps after spontaneous sIUFD with favorable outcome of the survivor (19).

Liu et al. (36) put forward a hypothesis: necrotic material from the deceased fetus may have been released into the shared amniotic cavity after the occurrence of sIUFD. A small amount of amniotic fluid containing this necrotic material could have entered the lungs of the surviving fetus, potentially leading to obstruction and the development of exudative lesions. These lesions likely went undetected during routine prenatal imageological examination. In future, more clinical studies need to be accumulated.

### Strengths and limitations

A major strength of our study is the relatively large sample size of twin pregnancies. We conducted a stratified analysis to compare prenatal outcomes of sIUFD in MC pregnancies according to the gestational age of single-fetal deaths.

However, our study has limitations that are important to consider when interpreting our results. There are no descriptions of long-term outcomes, neither in terms of anatomical-pathological confirmation on the TOP fetuses nor for the survivors with respect to postnatal outcomes, and this is a major limitation. The study's observational nature is a potential limitation, and the data were from a single center. The precise timing of death is usually indeterminate. Finally, given that our institution is a tertiary care referral center with high-risk pregnancies, the results of the study may not be applicable to all populations.

## Conclusions

Expectational treatment under close monitoring in MC twin pregnancies with sIUFD could be a good option for obtaining favorable outcomes. The greater the gestational age of sIUFD is, the higher the risk of brain injury for survival. The lowest risk of cerebral damage for the co-twin occurred before 25+6 weeks of gestation. There was a negative correlation between the number of gestational weeks at which a single fetus died and the number of delivery weeks in this study. In other words, the earlier the gestational age at which single fetal death occurred, the later the gestational age at delivery for survival co-twins. The prenatal outcomes of surviving co-twins with sIUFD before 24 weeks were better than those after 24 weeks. Finally, data analysis from our center revealed that favorable pregnancy outcomes may be useful for clinical consultations in the future.

## Data Availability

The datasets presented in this article are not readily available because, the datasets used and/or analyzed during the current study are available from the corresponding author upon reasonable request. Requests to access the datasets should be directed to Haiyan Yu, fanjy422@163.com.
